# Evaluation of the Reveal^®^ AST (SPECIFIC) for Antimicrobial Susceptibility Testing from Positive Blood Culture Spiked with Carbapenem-Resistant Isolates

**DOI:** 10.3390/pathogens13090722

**Published:** 2024-08-27

**Authors:** Delphine Girlich, Agnès B. Jousset, Cécile Emeraud, Inès Rezzoug, Reece Burwell, Pragya Singh, Paul A. Rhodes, Thierry Naas, Rémy A. Bonnin, Laurent Dortet

**Affiliations:** 1 Team “Resist” UMR1184 Immunology of Viral, Auto-Immune, Hematological and Bacterial Diseases (IMVA-HB), INSERM, Faculty of Medicine, Paris-Saclay University, 94270 Le Kremlin-Bicêtre, France; delphine.girlich@universite-paris-saclay.fr (D.G.); agnes.jousset@aphp.fr (A.B.J.);; 2 Bacteriology-Hygiene Unit, Assistance Publique-Hôpitaux de Paris, Bicêtre Hospital, 94270 Le Kremlin-Bicêtre, France; 3 Associated French National Reference Center for Antibiotic Resistance: Carbapenemase-Producing Enterobacterales, 94270 Le Kremlin-Bicêtre, France; 4 Specific Diagnostic, Mountain View, CA 94043, USA; 5 Next Gen Diagnostics, Cambridge CB10 1SA, UK; prhodes@nextgen-dx.com

**Keywords:** phenotypic antimicrobial susceptibility testing, blood culture, carbapenem-resistant Enterobacterales, rapid diagnostic

## Abstract

As bloodstream infections and associated septic shock are common causes of mortality in hospitals, rapid antibiotic susceptibility testing (AST) performed directly on positive blood cultures is needed to implement an efficient therapy in clinical settings. We evaluated the Reveal^®^ rapid AST system on a collection of 197 fully characterized carbapenem-resistant Enterobacterales, including 177 carbapenemase producers (CPE) spiked in blood culture bottles. The clinical categorization based on the Minimal Inhibitory Concentration (MIC) determination of eighteen antimicrobial molecules was compared to the clinical categorization based on the disk diffusion assay as a reference. The Reveal AST system provided results within a mean time to result of 5 h. Overall, the categorical agreement (CA) between the two techniques was 94.1%. The rates of very major errors (VMEs), major errors (MEs) and minor errors (mEs) were 3.8%, 3.7% and 5.6%, respectively. Imipenem was the antimicrobial with the lowest CA rate (78.7%), with rates of 15% VMEs and 10.7% MEs, but the performances were better when considering only the non-CPE category (CA of 89%). On this resistant collection of Enterobacterales with numerous acquired β-lactamases, the Specific Reveal assay proved to be useful for a rapid determination of AST compatible with a quick adaptation of the patient’s antimicrobial treatment.

## 1. Introduction

Sepsis represents a critical condition wherein organ dysfunction arises from an unbalanced host reaction to infection, posing a significant worldwide health concern with regional disparities related to patient care and access to rapid diagnostic techniques [1]. More than 1.2 million episodes of bloodstream infections (BSIs) are estimated to occur each year in Europe leading to 157,000 deaths [2].

In a global context of increasing drug resistance and concurrent risk of therapeutic failure, early identification and appropriate management in the initial hours after the development of sepsis improve outcomes [3]. Indeed, infections caused by multi-drug resistant (MDR) Gram-negative bacilli are associated with higher mortality rates, longer hospitalizations and increased healthcare costs partially due to inadequate initial therapy [4,5,6].

The recent development of rapid and easy-to-use molecular biology-based tests has proved their usefulness for better patient care in terms of length of stay and cost per day of hospitalization [7]. However, regarding Gram-negative pathogens, these techniques can detect only targeted genes encoding extended spectrum β-lactamase (ESBL) and carbapenemases. While PCR-based methods remain costly, immunochromatography-based detection kits offer a more affordable alternative, though they do not address the issue of identifying unknown resistance mechanisms [8,9]. Culture-based phenotypic methods remain the gold standard and provide information on multiple antimicrobial families including β-lactams, fluoroquinolones, aminoglycosides and sulphonamides.

Traditional antimicrobial susceptibility testing (AST) methods (broth microdilution and disk diffusion) or semi-automated commercial methods (MicroScan™, BD Phoenix™, Vitek2™, Sensititre™) require at least 16 h to obtain the antibiogram, and some of them require grown colonies which add another 16 h to 24 h delay. In that context, several automated systems have been developed these past years to reduce the turnaround time to obtain AST directly from positive blood cultures in less than 12 h [10,11].

Among them, the Reveal^®^ AST system (Specific Diagnostic, USA) uses an innovative sensor technology to detect the growth of bacterial populations via their emission of volatile organic compounds (VOCs) during growth [12,13,14] using standard commercially available 96-well dried antibiotic plates (Microscan; Beckman Coulter). Growth is imaged by sensors every 10 min to monitor the change in intensity over time.

Data on the performances of the Reveal AST system on a large collection of MDR Enterobacterales are lacking. Here, 197 well-characterized carbapenem-non-susceptible Enterobacterales (CREs), including diverse carbapenemase-producing Enterobacterales (CPEs), representative of French epidemiology, were spiked in negative blood culture, incubated and tested with Reveal^®^ AST.

## 2. Material and Methods

### 2.1. Bacterial Isolates

A total of 197 Enterobacterales with resistance or decreased susceptibility to carbapenems (CREs), sent to the French National Reference Center (f-NRC) for antimicrobial resistance, were included in the study (Table 1, Table 2 and Table S1). These isolates were representative of French and European epidemiology. This collection was made of OXA-48-like producers (n = 88), NDM producers (n = 26), VIM producers (n = 15), KPC producers (n = 10), IMP producers (n = 5), producers of other rare class A carbapenemases (IMI-type, NMC-A, GES-5) (n = 5) and isolates producing at least 2 carbapenemases (n = 28). Non-carbapenemase-producing CREs (CRE non-CPE) (n = 20) were also included. Non-CPE specimens comprised ESBL and/or AmpC producers with decreased permeability to carbapenems. Two specific variants with no carbapenemase activity were also included: KPC-39 and OXA-163. Whole Genome Sequencing using Illumina’s technology was performed on all isolates to ensure their resistome. The specific variants of β-lactamases produced by those isolates are summarized in Table S1.

### 2.2. Spiking of Culture Blood Bottles

Challenge strains were cultured overnight on Mueller–Hinton agar. Bottles containing 10 mL of sterile human blood were spiked with 1 mL of a serially diluted to 10^−6^ 0.5 McFarland suspension to achieve a final concentration of 10^2^ CFU per bottle. The inoculated bottles were incubated in a BacT/ALERT^®^ 3D automate (BioMérieux^®^, Marcy l’Etoile, France) until positive results were detected. Each sample was then assayed on the Reveal^®^ AST System. Positive blood cultures with more than 16 h post-positive detection were excluded from the study.

### 2.3. Antimicrobial Susceptibility Testing

The Reveal system provided results for 18 molecules that were also tested by disk diffusion on Mueller–Hinton agar (Biorad, Marnes la Coquette, France). Tested disks (i2a, Sceaux, France) were as follows: amikacin 30 µg (AMK30), ampicillin 20 µg (AM20) on *E. coli* isolates, aztreonam 30 µg (ATM30), cefepime 30 µg (FEP30), cefotaxime 5 µg (CTX5), cefoxitin 30 µg (FOX30) on *E. coli* and *K. pneumoniae* isolates, ceftazidime 10 µg (CAZ10), ceftazidime/avibactam 10 µg + 4 µg (CZA), ciprofloxacin 5 µg (CIP5), ertapenem 10 µg (ETP10), gentamicin 10 µg (GMN10), imipenem 10 µg (IPM10), levofloxacin 5 µg (LEV5), meropenem 10 µg (MEM10), piperacillin 30 µg (PRL30), piperacillin/tazobactam 36 µg (TZP36), tobramycin 10 µg (TOB10) and trimethoprim/sulfamethoxazole 25 µg (SXT25).

Inhibition disk diameters were read after 16 h–24 h of incubation, and clinical categorizations were determined according to the guidelines provided by CASFM-EUCAST 2023 [15].

Noticeably, the Reveal software version at that time provided ampicillin and cefoxitin results only for *E. coli.* Piperacillin/tazobactam and ceftazidime/avibactam results were not provided for *K. aerogenes* and *C. koseri*, respectively.

For discordant results between Reveal System and disk diffusion categorization, broth microdilution using Sensititre plates were used as reference (GN4F, GN6F and ESB1F panels).

### 2.4. The Reveal Technology

An array of 7-indicator nanoporous printed volatile sensor arrays are positioned above each well of a 96-well antibiotic plate. Changes in sensor color produced by the volatile emissions associated with microbial growth are used to assay population growth and, hence, antimicrobial effectiveness in each well. Sensor color is monitored every 10 min via line scan, producing 21 plots (7 sensors x R, G and B color channels). The Reveal system includes an expert system that categorizes each antibiotic according to updated EUCAST recommendations.

### 2.5. Quality Control (QC)

*Escherichia coli* ATCC 25922, *Pseudomonas aeruginosa* ATCC 27853 and *Klebsiella pneumoniae* (SHV-18, ESBL producer) ATCC 700603 were used as controls. QC was performed by inoculation of 125 µL of a 0.5 McFarland suspension into 25 mL of Pluronic water and then inoculation of 115 µL of this suspension in each well of the plate by using the MicroScan Renok device (Beckman Coulter). The Reveal instrument on which the QC testing was performed rotated every week so that each unit was controlled every 3 weeks. Reveal sealer QC was also performed each week.

### 2.6. Data Analysis

For each strain/antimicrobial combination, clinical categorization generated from the disk diffusion method was compared to the categorization generated by the Reveal system. CA-SFM-EUCAST v2023 interpretive guidelines were used for determination of the susceptible at standard exposure (S), susceptible at increased exposure (I), or resistant (R) categories.

For each antimicrobial agent, the rates of categorical agreement (CA), very major (VME) major (ME) and minor (mE) errors were calculated. Essential agreement (EA) was not determined due to the study design. Indeed, only clinical categorizations could be compared between both techniques (disk diameter versus MIC).

VME corresponded to the case where the Sensititre reference result was R (resistant) and the Reveal result was S (susceptible at standard exposure) or I (susceptible at increased exposure). ME corresponded to the case where the Sensititre result was S or I, and the Reveal result was R. Lastly, minor error (mE) was defined as the case where the Reveal indicated the strains as I while the reference method showed either susceptibilities, or vice versa.

To analyze ME and VME discrepancies, commercialized broth microdilution plates (Sensititre) were used to reach a consensus reference.

The time to result (TTR) for each antibiotic from Reveal system was determined.

## 3. Results

### 3.1. Time to Result

Using the Reveal system, AST results were available in an average of 5.0 h across all strains and antibiotics, with each antibiotic averaging between 3.0 and 8.0 h (Table S2). The TTR analysis revealed large disparities between tested molecules. Ampicillin was the quickest result obtained (mean of 3 h). However, in our collection, all tested strains were resistant to this antibiotic. Most of ertapenem, imipenem and meropenem results were available after a delay of 6 h 30 min (Table S2).

In our routine workflow, this short time to result will be interesting for a blood culture that flags Enterobacterales-positive between 00:00 and 10:00 a.m. (day 0); the AST on standard MH will be interpreted by a microbiologist the following day (day 1), whereas with the Reveal technology, an AST result will be obtained the same day (day 0).

When a blood culture flags positive between 10:00 a.m. and 3:00 p.m. (day 0), the Reveal AST will be available by the end of the day 0, but it is likely that the antimicrobial therapy will not be adapted by the physician before the next morning (day 1). In that specific case, the Reveal System does not demonstrate superiority to save time in comparison with disk diffusion on MH plates.

### 3.2. Analytical Performances

Eighteen molecules on 197 isolates comprising seven different species were tested with the Reveal system and compared to AST performed via disk diffusion leading to a total of 3271 strain–drug measurements. All results are indicated in Table S3.

Overall, the Reveal system achieved a 94.1% categorical agreement (CA), ranging from 78.7% for imipenem to 100% for ampicillin (Table 3). Of note, all tested isolates were resistant to ampicillin.

Based on the CA values, the performance of Reveal was excellent for piperacillin (99.4%), gentamicin (94.4%), amikacin (98.9%), ceftazidime (97.9%), ceftazidime/avibactam (97.4%), tobramycin (96.9%), levofloxacin (96.9%), cefotaxime (96.4%) and cotrimoxazole (95.9%).

The performance for ciprofloxacin susceptibility testing was lower, with a CA at 91.9%. However, among the six observed MEs, five disk diameter measurements were within the area of technical uncertainty.

Compared to disk diffusion, 186 errors (5.7%) occurred with the Reveal AST system. Among them, 64 were mEs, 39 MEs and 83 VMEs. Most of the VMEs involved piperacillin/tazobactam (n = 13), cefepime (n = 13), imipenem (n = 11) and meropenem (n = 10).

Regarding the analysis of piperacillin/tazobactam VMEs (n = 13/186, 7%), only one diameter was affected by the area of technical uncertainty (19 mm) which was confirmed by Sensititre MIC (16 mg/L), where Reveal led to a firm susceptible categorization (MIC <= 8 mg/L). Among the 13 isolates, 6 were OXA-48 producers, 3 were IMP-producers, 2 were VIM-producers and 2 isolates did not produce any carbapenemase.

Regarding the analysis of VMEs linked to cefepime (13/146, 8.9%), 12 isolates were categorized as susceptible to increased exposure (I) by the Reveal system and resistant by disk diffusion: one IMP-, one NDM-, one OXA-48-like-, four KPC-, four VIM- and two OXA-48-like+VIM-producers.

### 3.3. Results for Carbapenems

A particular focus on the three tested carbapenems was made. It is important to consider differently the CPEs and non-CPEs within this collection. Indeed, carbapenems are commonly used to treat infections caused by ESBL or AmpC producers when MICs remain below the clinical breakpoint. But when a carbapenemase is produced and if the isolate is categorized as fully susceptible (S) or susceptible at increased exposure (I), a carbapenem-based treatment should be used with caution and, if possible, in combination with another antimicrobial according to CASFM-EUCAST 2023 guidelines [16,17].

CA was the lowest for imipenem (78.7%), corresponding either to false susceptibilities (11 VME/71, 15.5%) or false resistance (11 ME/106, 10.4%) in equal proportion. CA was higher (89%) when considering only the non-CPE category (n = 20). CA was the highest for ertapenem (95.5%) and reached 100% non-CPE CRE, but our collection was made of only 21/197 ertapenem susceptible strains (13 OXA-48-like producers, 4 VIM-1 producers, 3 IMP-producers and the KPC-39 producer that had no carbapenemase activity).

Oppositely, for meropenem, the CA was of 87.8% on the whole collection with 67% for non-CPE CREs and 90% for CPEs. For both ertapenem and meropenem, VMEs represented most of the discrepancies. The Reveal system tended to underestimate the MIC of both antibiotics, leading to an overestimation of their respective susceptibility rate.

All errors involving carbapenems with details about the associated isolates are listed in Table S4.

## 4. Discussion

This study was designed to challenge the Reveal System on a specific collection made of carbapenem-resistant Enterobacterales with various enzymes for various phenotypic features, which does not mimic a real-life clinical microbiology laboratory. One limitation of this study is the comparison of clinical categorization obtained by Reveal (based on a MIC value) to the clinical categorization obtained by a disk diffusion assay (based on diameters). Accordingly, the essential agreement of Reveal was not assessed with this protocol.

Interestingly the value of CA for ertapenem was the highest of the three carbapenems. This indicates that ertapenem could be the most sensitive carbapenem for the detection of carbapenem-resistant isolates. However, as previously reported, this marker is not sufficient to distinguish CPEs from other CREs, particularly in countries where OXA-48-like producers are the most prevalent carbapenemases [18]. The Reveal system would benefit from the implementation of an expert system that could predict the presence or absence of a carbapenemase based on multiple markers, as it is used for other automated antimicrobial susceptibility testing methods (e.g., VITEK from bioMérieux, Microscan from Beckman-Coulter or Phoenix from Becton-Dickinson, New York, NY, USA).

The first clinical evaluation of the Reveal rapid AST system showed a CA and VME rate of 96.3% and 1.3%, respectively [14]. This study included 104 randomly selected blood cultures with Gram-negative organisms obtained from one urban hospital with various resistance profiles but no CPEs [14]. To enlarge their resistant collection, Tibbetts et al. also tested spiked blood culture with a small number (n = 33) of “highly resistant” isolates. However, data regarding the proportion of each carbapenemase (KPC, VIM, IMP, NDM or OXA-48) were lacking.

Another study, designed to evaluate the performance of the Reveal rapid AST with 317 carbapenem-resistant organisms from the CDC AR isolate bank, showed a categorical agreement of 90.9%, 94.5% and 91.5%, respectively, for meropenem, ertapenem and imipenem [19]. Those results were higher than those obtained in our study for meropenem and imipenem especially (CA of 87.8% and 78.7%, respectively). Again, the detailed composition of carbapenemases was not available. Since OXA-48 producers possess a lower hydrolytic activity compared to KPC or metallo-β-lactamases, a higher proportion of those OXA-48-like producers in Europe compared to the USA might explains these differences.

A drawback of the Reveal system is that some Enterobacterales species such as *Morganella morganii*, *Proteus mirabilis*, *Raoultella ornithinolytica*, *Salmonella enterica*, *Serratia marcescens* and *Hafnia alvei* were not included in the database at the time the study was conducted. A future update will likely implement these additional species, which correspond to approximately 8% of bacteremia caused by to Enterobacterales occurring in our hospital.

Regarding the tested panel, it would be clinically relevant to include some additional antibiotics such as polymyxins, tigecycline and fosfomycin, which may remain effective against some CPEs. Mecillinam is also missing from the panel, and yet is an effective molecule that proved activity in vitro on most of *E. coli* and *K. pneumoniae* isolates that produce OXA-48 or NDM-like enzymes [20]. This molecule, which was recently approved in 2024 by the FDA for treating uncomplicated urinary tract infections, might be a relevant therapeutic option for the treatment of bacteremia originating from urinary sources.

Finally, for countries with a high prevalence of bacteriemia associated with CREs, it seems relevant to add several b-lactam/b-lactamase inhibitor combinations (such as ceftolozane/tazobactam, imipenem/relebactam, meropenem/vaborbactam) as well as cefiderocol in the panel to increase the number of therapeutical options. Furthermore, as already reported for the disk diffusion method, the analysis of susceptibility results of several antimicrobial markers by an informatic expert system might also help to detect and characterize the carbapenem resistance mechanism, and even identify the carbapenemase type in some cases [18,21].

## 5. Conclusions

The Reveal AST system addresses the clinical need for rapid susceptibility testing directly from Gram-negative blood cultures, while at the same time reducing the cost per test in comparison with molecular assays. It delivers phenotypic antimicrobial susceptibility testing with MIC results for a large panel of antimicrobials (including different families and narrow- to broad-spectrum drugs) in an average of 5 h. Reveal AST proved to be useful for a rapid determination of AST, compatible with a quick adaptation of the patient’s antimicrobial treatment even in the case of multi-drug-resistant bacteria.

Of note, the data and analysis used in this study were conducted using an outdated version of the instrument, software, panel and algorithm, which might not accurately reflect the system’s current capabilities in the field.

A further prospective clinical study is needed to inform on the potential gain for the patient’s management, notably in terms of antimicrobial therapy adaptation, clinical outcome and hospitalization duration.

## Figures and Tables

**Table 1:** Study set details.

Number of species		7
Number of antimicrobials		18
Number of strains		197
Number of CPE		
	OXA-48-like	88
	KPC	10
	NDM	26
	VIM	15
	IMP	5
	Multiple carbapenemases	28
	Rare carbapenemases	5
Number of non-CPE CRE		20

**Table 2:** Susceptibility categorization of tested strains.

	Disk Diffusion	Reveal
Number of total strain–drug pairs	3348	3272
Number of S strain–drug pairs	1051	1060
Number of I strain–drug pairs	94	127
Number of R strain–drug pairs	2203	2085

**Table 3:** Performance of the Reveal AST system (18 antimicrobial tested on 197 carbapenem-non-susceptible Enterobacterales).

Antimicrobials	Reveal AST Results				Number of		
	S	I	R	CA %	Very Major Errors (%)	Major Errors (%)	Minor Errors (%)
Ampicillin ^a^	0	NA	54	100%	0/37 (0%)	0/18 (0%)	NA
Piperacillin	4	NA	192	99.5%	1/194 (0.5%)	0/3 (0%)	NA
Piperacillin/Tazobactam	19	NA	173	93.2%	13/186 (7%)	0/6 (0%)	NA
Cefoxitin ^a^	27	NA	28	85.5%	8/36 (22%)	0/19 (0%)	NA
Cefotaxime	27	0	170	96.4%	0/167 (0%)	3/25 (12%)	4/30 (13.3%)
Ceftazidime	31	8	157	98.0%	4/161 (2.5%)	0/31 (0%)	0/35 (0%)
Ceftazidime/Avibactam	127	NA	66	97.4%	5/71 (7%)	0/125 (0%)	NA
Cefepime	44	19	134	91.4%	13/146 (9%)	1/43 (2.3%)	3/51 (5.9%)
Aztreonam	51	7	139	90.9%	0/128 (0%)	11/59 (18.6%)	7/69 (10.1%)
Imipenem	88	38	71	78.7%	11/71 (15.5%)	11/106 (10.4%)	20/126 (15.6%)
Ertapenem	27	NA	170	95.4%	8/177 (4.5)	1/20 (5%)	NA
Meropenem	109	36	51	87.8%	10/61 (16.4%)	1/101 (1%)	12/136 (8.8%)
Amikacin	157	NA	40	99.0%	2/42 (4.8%)	0/155 (0%)	NA
Gentamicin	99	NA	98	99.5%	0/96 (0%)	1/100 (1%)	NA
Tobramycin	70	NA	127	97.0%	4/129 (3.1%)	2/68 (2.9%)	NA
Ciprofloxacin	39	12	146	91.9%	0/140 (0%)	6/50 (12%)	10/57 (17.5%)
Levofloxacin	63	3	131	96.9%	2/132 (1.5%)	1/61 (1.6%)	3/64 (4.7%)
Trimethoprim/ Sulfamethoxazole	59	4	134	95.9%	2/135 (1.5%)	1/60 (1.7%)	5/62 (8.1%)

^a^ Ampicillin and cefoxitin were tested only on *E. coli*. S = susceptible at standard dosage. I = susceptible at increased exposure. R = resistant. CA, categorical agreement.

## Data Availability

The original contributions presented in the study are included in the article/supplementary material, further inquiries can be directed to the corresponding author/s.

## Supplementary Materials

The following supporting information can be downloaded at: https://www.mdpi.com/article/10.3390/pathogens13090722/s1, Table S1: The specific variants of β-lactamases produced by isolates included in the study; Table S2: TTR_analysis; Table S3: Result of all Enterobacterales tested (n = 197); Table S4: Analysis of errors involving carbapenems.

## References

[1] Rudd K.E., Johnson S.C., Agesa K.M., Shackelford K.A., Tsoi D., Kievlan D.R., Colombara D.V., Ikuta K.S., Kissoon N., Finfer S. (2020). Global, Regional, and National Sepsis Incidence and Mortality, 1990-2017: Analysis for the Global Burden of Disease Study. Lancet.

[2] Goto M., Al-Hasan M.N. (2013). Overall Burden of Bloodstream Infection and Nosocomial Bloodstream Infection in North America and Europe. Clin. Microbiol. Infect..

[3] Evans L., Rhodes A., Alhazzani W., Antonelli M., Coopersmith C.M., French C., Machado F.R., Mcintyre L., Ostermann M., Prescott H.C. (2021). Surviving Sepsis Campaign: International Guidelines for Management of Sepsis and Septic Shock 2021. Intensive Care Med..

[4] Daikos G.L., Markogiannakis A., Souli M., Tzouvelekis L.S. (2012). Bloodstream Infections Caused by Carbapenemase-Producing Klebsiella Pneumoniae: A Clinical Perspective. Expert. Rev. Anti Infect. Ther..

[5] Paoli C.J., Reynolds M.A., Sinha M., Gitlin M., Crouser E. (2018). Epidemiology and Costs of Sepsis in the United States-An Analysis Based on Timing of Diagnosis and Severity Level. Crit. Care Med..

[6] Eubank T.A., Long S.W., Perez K.K. (2020). Role of Rapid Diagnostics in Diagnosis and Management of Patients with Sepsis. J. Infect. Dis..

[7] Mponponsuo K., Leal J., Spackman E., Somayaji R., Gregson D., Rennert-May E. (2022). Mathematical Model of the Cost-Effectiveness of the BioFire FilmArray Blood Culture Identification (BCID) Panel Molecular Rapid Diagnostic Test Compared with Conventional Methods for Identification of Escherichia Coli Bloodstream Infections. J. Antimicrob. Chemother..

[8] Bernabeu S., Ratnam K.C., Boutal H., Gonzalez C., Vogel A., Devilliers K., Plaisance M., Oueslati S., Malhotra-Kumar S., Dortet L. (2020). A Lateral Flow Immunoassay for the Rapid Identification of CTX-M-Producing Enterobacterales from Culture Plates and Positive Blood Cultures. Diagnostics.

[9] Takissian J., Bonnin R.A., Naas T., Dortet L. (2019). NG-Test Carba 5 for Rapid Detection of Carbapenemase-Producing Enterobacterales from Positive Blood Cultures. Antimicrob. Agents Chemother..

[10] Grohs P., Rondinaud E., Fourar M., Rouis K., Mainardi J.-L., Podglajen I. (2020). Comparative Evaluation of the QMAC-dRAST V2.0 System for Rapid Antibiotic Susceptibility Testing of Gram-Negative Blood Culture Isolates. J. Microbiol. Methods.

[11] Zalas-Więcek P., Bogiel T., Gospodarek-Komkowska E. (2022). The Accelerate Pheno^TM^ System—A New Tool in Microbiological Diagnostics of Bloodstream Infections: A Pilot Study from Poland. Pathogens.

[12] Lim S.H., Mix S., Xu Z., Taba B., Budvytiene I., Berliner A.N., Queralto N., Churi Y.S., Huang R.S., Eiden M. (2014). Colorimetric Sensor Array Allows Fast Detection and Simultaneous Identification of Sepsis-Causing Bacteria in Spiked Blood Culture. J. Clin. Microbiol..

[13] Lonsdale C.L., Taba B., Queralto N., Lukaszewski R.A., Martino R.A., Rhodes P.A., Lim S.H. (2013). The Use of Colorimetric Sensor Arrays to Discriminate between Pathogenic Bacteria. PLoS ONE.

[14] Tibbetts R., George S., Burwell R., Rajeev L., Rhodes P.A., Singh P., Samuel L. (2022). Performance of the Reveal Rapid Antibiotic Susceptibility Testing System on Gram-Negative Blood Cultures at a Large Urban Hospital. J. Clin. Microbiol..

[15] The European Committee on Antimicrobial Susceptibility Testing Breakpoint Tables for Interpretation of MICs and Zone Diameters, Version 13.1, 2023. http://www.eucast.org/clinical_breakpoints/.

[16] Mimoz O., Grégoire N., Poirel L., Marliat M., Couet W., Nordmann P. (2012). Broad-Spectrum β-Lactam Antibiotics for Treating Experimental Peritonitis in Mice Due to Klebsiella Pneumoniae Producing the Carbapenemase OXA-48. Antimicrob. Agents Chemother..

[17] (2024). CA-SFM Comité de l’Antibiogramme de La Société Française de Microbiologie (CA-SFM)—Recommandations. https://www.sfm-microbiologie.org/wp-content/uploads/2023/06/CASFM2023_V1.0.pdf.

[18] Duque M., Bonnin R.A., Dortet L. (2024). Evaluation of the French Novel Disc Diffusion-Based Algorithm for the Phenotypic Screening of Carbapenemase-Producing Enterobacterales. Clin. Microbiol. Infect..

[19] Rajeev L., Khomtchouk K., Singh P., Rhodes P.A. (2021). Detection of Carbapenem Resistance by the Reveal Rapid Phenotypic AST Assay in Strains from the CDC and FDA Antimicrobial Resistant Isolate Bank.

[20] Emeraud C., Godmer A., Girlich D., Vanparis O., Mahamdi F., Creton E., Jousset A.B., Naas T., Bonnin R.A., Dortet L. (2022). Activity of Mecillinam against Carbapenem-Resistant Enterobacterales. J. Antimicrob. Chemother..

[21] Dortet L., Cuzon G., Plésiat P., Naas T. (2016). Prospective Evaluation of an Algorithm for the Phenotypic Screening of Carbapenemase-Producing Enterobacteriaceae. J. Antimicrob. Chemother..

